# Length of Lecture Terms

**Published:** 1847-08

**Authors:** 


### LENGTH OF LECTURE TERMS.

One of the recommendations of the late Medical Convention was, that the sessions of our medical schools should be extended from four months to six. From their advertisement, just sent abroad, we see that the Medical Faculty of the University of Pennsylvania have accepted this proposition so far as to commence the session on the 18th of October and continue it to the end of March. It is proper that the oldest medical school in the country should thus take the lead in this matter, and it may be expected that the other schools will follow her example. Perhaps so great an extension as two months would not be advisable at once, but it may be brought about gradually, so as in a few years to establish six months as the duration of the course. When this comes to be the case, we may hope that students will go to the schools with a larger stock of patience, and be able to hold out for at least the four months which they have professed heretofore to give to lectures. As it is now, too many are contented with three months attendance, and not a few run away at the end of the second. We have heard of some, in times past, who were "armed and equipped" with lancet and drugs, and, sign-board painted, were ready to take a boat by the 8th of January, "the world all before them where to choose." This has been one of the sins against which medical teachers have hitherto in vain lifted up their voices. A lengthened term will do something towards correcting it; an enlightened public sentiment would do much more.

